# Combining Phototherapy and Gold-Based Nanomaterials: A Breakthrough in Basal Cell Carcinoma Treatment

**DOI:** 10.3390/ijms252111494

**Published:** 2024-10-26

**Authors:** Karolyne Silva Baioco, Raquel Pereira, Tânia Ferreira-Gonçalves, João M. P. Coelho, Maria Manuela Gaspar, Catarina Pinto Reis

**Affiliations:** 1 Research Institute for Medicines (iMed.ULisboa), Faculty of Pharmacy, Universidade de Lisboa, Av. Professor Gama Pinto, 1649-003 Lisboa, Portugal; silvabaiocokarolyne@gmail.com (K.S.B.); ana.raquel1705@gmail.com (R.P.); tsgoncalves@ciencias.ulisboa.pt (T.F.-G.); manuelagaspar@campus.ul.pt (M.M.G.); 2 Instituto de Biofísica e Engenharia Biomédica (IBEB), Faculdade de Ciências, Universidade de Lisboa, Campo Grande, 1749-016 Lisboa, Portugal; jmcoelho@ciencias.ulisboa.pt

**Keywords:** skin cancer, basal cell carcinoma, photothermal therapy, gold nanoparticles

## Abstract

Basal cell carcinoma (BCC) is the most common type of skin carcinoma worldwide. BCC development is the result of a complex interaction between environmental, phenotypic, and genetic factors. While conventional treatments such as surgery and topical therapies have demonstrated variable efficacy (some of them with limited efficacy), they are not free of adverse side effects, most of them debilitating. Thus, there is a notable gap in the literature regarding alternative and non-invasive therapeutic options. This review aims to address this gap, exploring the potential of photothermal therapy (PTT) combined with metallic nanoparticles, namely gold nanoparticles (AuNPs), as a minimally invasive treatment approach. Through a comprehensive review of the literature in the period from 2014 to 2024, using experimental investigations, this review seeks to elucidate the intricate interplay between genetic factors, environmental influences, and the tumor microenvironment in BCC disease progression, with PTT as a potential therapeutic strategy. Those studies confirmed an enhanced targeting of cancer cells and selective ablation of tumor tissue, using emerging technologies like PTT. A significant tumor reduction, often exceeding 50%, was observed, with some studies reporting complete elimination of the tumor. The main adverse effects noted were localized skin irritation and transient hyperpigmentation, but these were generally minimal and manageable, highlighting the promise of PTT as an effective treatment. Thus, by leveraging the unique properties of AuNPs to enhance the effectiveness of PTT, the targeting of cancer cells can more precisely occur, reducing collateral damage to healthy tissues. This approach not only aims to achieve better clinical results, but also contributes to the broader knowledge base in the field of BCC research. Continued research and clinical trials will be crucial in refining those techniques and validating their efficacy, ultimately paving the way for more effective and less invasive treatments for BCC.

## 1. Background

The skin comprises stratified layers that serve as a multifunctional barrier and contribute significantly to homeostasis [1,2,3]. Understanding the intricate cellular composition of the skin is important to elucidate pathophysiological mechanisms underlying dermatological conditions like basal cell carcinoma (BCC) [3].

**Figure 1:**
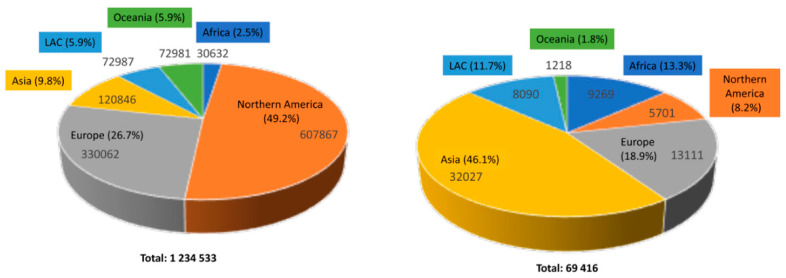
Estimated numbers of new skin non-melanoma cases and deaths worldwide in 2022 for both sexes and all ages: incidence (**left**) and mortality (**right**). Data adapted from the Globocan Cancer Observatory by IARC [9].

BCC is the most common human skin cancer, with roughly 3.6 million cases diagnosed each year [4,5]. While often less aggressive than other types of skin cancer, such as melanoma, BCC remains a major challenge due to its rising occurrence and impact on quality of life, emerging as a global public health concern [6]. In fact, BCC represents the most widespread form of skin cancer among individuals with fair skin, with an estimated lifetime risk of pathology occurrence in this demographic being approximately 30% [7,8]. Although the global incidence of BCC is steadily rising (Figure 1), accurate estimations are challenging due to inconsistent registration practices.

Despite extensive research on BCC, a notable gap in the literature regarding non-invasive and more effective therapies persists, and there are some reasons that can explain this gap.

While previous studies have clarified the role of UV-B radiation exposure, genetic factors, and aberrant hedgehog signaling in BCC development, there is still limited knowledge about the complex interaction between these factors and their contribution to tumor progression [10]. Furthermore, current therapeutic approaches for BCC, including surgery and topical treatments, often exhibit limitations, such as incomplete tumor removal or adverse side effects, highlighting the need for more targeted and effective treatment strategies [11].

Therefore, this review aims to address this gap by investigating the prime molecular pathways driving BCC pathogenesis and evaluating the potential of phototherapy with nanoparticles as a minimally invasive treatment option [12]. Through a comprehensive analysis of the literature and experimental investigations, this overview seeks to provide valuable insights into the mechanisms underlying BCC development and identify innovative treatment interventions that can improve patient outcomes and quality of life [4,5].

## 2. Methodology

This literature review was conducted over a period spanning the last ten years, from 2014–2024. This timeframe was chosen to ensure the inclusion of the most recent and relevant studies in the field of BCC and its treatment modalities.

The primary database utilized for the literature search was PubMed, a comprehensive and widely used resource for biomedical and health-related literature. PubMed was selected for its extensive collection of peer-reviewed articles, clinical studies, and reviews, ensuring a thorough and high-quality source of information. “Basal cell Carcinoma”; “BCC”; “Photothermal therapy”; “Gold nanoparticles”; “Chemotherapy”; “Photodynamic therapy”; “Hedgehog pathway”; “PTCH1 gene”; “SMO gene”; “TP53 gene”; and “Mohs micrographic surgery” were the keywords used.

## 3. Clinical Manifestations, Risk Factors, Diagnosis and Treatment of BCC

**Figure 2:**
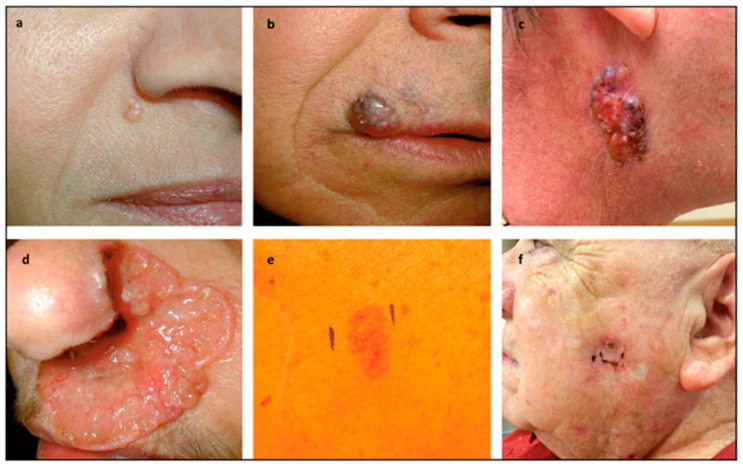
(**a**) Nodular Basal Cell Carcinoma: Characterized by a pearly surface and telangiectasias, typically located lateral to the right alar crease. (**b**) Pigmented Variant: This form presents above the right upper lip and extends past the vermilion border into the lip, showing distinct pigmentation. (**c**) Large Nodular Basal Cell Carcinoma: Notable for its size, these variant features characteristic telangiectasias and pigmented areas, commonly found on the right side of the neck. (**d**) Ulcerated Aggressive Basal Cell Carcinoma (“Rodent Ulcer”): This severe form spreads locally, causing extensive destruction, as seen in the left nasal ala of the nose. (**e**) Superficial Basal Cell Carcinoma: Appearing as a red patch, this variant typically manifests on the trunk. (**f**) Recurrent Basal Cell Carcinoma: This form appears at the site of a previous electrodessication and curettage (EDC), indicating recurrence of the carcinoma. Reprinted with permission from reference [16].

BCC exhibits a spectrum of clinical and histological presentations, reflecting its multifaceted nature and diverse origins, as represented in Figure 2.

Characterized by papules or nodules with telangiectasia, nodular BCC often poses a diagnostic challenge due to its varied morphological features [13,14]. Superficial BCC appears as flat, scaly lesions with clear borders, often on the torso of younger adults. Despite its mild appearance, it can extend beneath the surface, needing regular monitoring and diagnosis [13,14]. Morpheaform BCC, though less frequent, represents a distinctive histological variant characterized by scar-like plaques with indistinct borders, resembling localized scleroderma [15]. This subtype often exhibits subclinical spread and extensive local tissue destruction, emphasizing the importance of early detection and intervention [13,14].

Among the common subtypes, nodular BCC stands out as the most prevalent, representing most cases encountered clinically.

BCC typically manifests after the age of 50, with the prevalence in females being two times greater compared to males. However, cases may occur earlier, particularly in individuals with genetic predisposition syndromes like xeroderma pigmentosum (XP) or basal cell naevus syndrome (BCNS), who may develop BCC before the age of 20 [14,16,17].

Prolonged UV-B exposure, especially in childhood, is a major risk factor, causing reactive oxygen species (ROS) production and mutations in the p53 tumor suppressor gene [4,18]. Intermittent and intense UV exposure is the leading environmental risk factor for BCC. Incidence correlates with proximity to the equator, reflecting higher UV levels. Tanning beds, photosensitizing drugs, and ionizing radiation further increase BCC risk [16,18].

Additionally, genes are central to the etiology and progression of BCC [10]. Genetic factors and hereditary predispositions, including aberrant hedgehog signaling, contribute significantly to BCC pathogenesis [10]. Syndromes like Gorlin syndrome (GS), xeroderma pigmentosum, and Bazex syndrome increase BCC risk [4,10,15]. GS, caused by mutations in the PTCH1 gene on chromosome 9q22.3–q316, predisposes individuals to multiple BCCs by disrupting the sonic hedgehog pathway [15].

**Figure 3:**
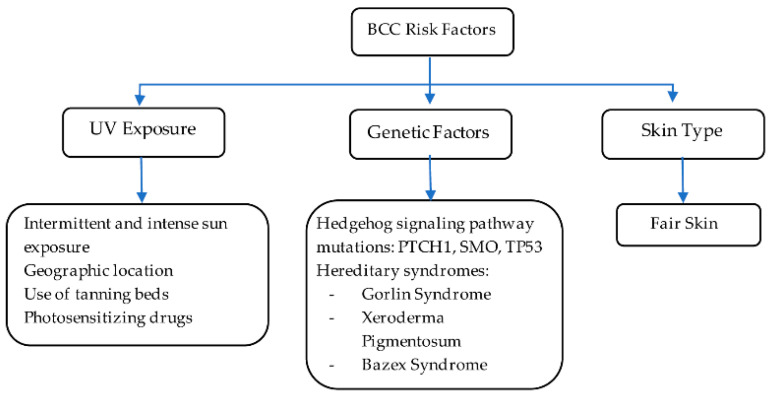
BCC risk factors. Information adapted from references [4,10,16,17,18,19].

In sporadic BCC cases without hereditary factors, the genetic landscape is not fully understood. However, mutations in the hedgehog (Hh) pathway are found in about 85% of these BCCs, primarily involving PTCH1 (70–75%), SMO (10–20%), and TP53 (40–65%). PTCH1 mutations disrupt the sonic hedgehog signaling, while TP53 mutations impair the p53 protein’s function, promoting BCC growth. Understanding these genetic changes is essential for developing targeted therapies to mitigate tumor progression [19]. Skin type plays a significant role in the risk and development of BCC. According to the literature, Fitzpatrick skin types I (very fair skin) and II (fair skin) are more likely to develop BCCs, with a lifetime risk of 30% [20]. As highlighted below in Figure 3, key environmental and genetic factors contribute to BCC pathogenesis, illustrating the interplay between UV exposure, genetic mutations, and hereditary syndromes.

A potential correlation between the onset of BCC and a dysembryogenetic process has long been hypothesized, i.e., the development of this type of skin cancer could be linked to errors or abnormalities during embryonic development [21,22,23,24].

Diagnosis of BCC relies on clinical and dermatoscopic examination, with characteristic features including arborizing telangiectasias, leaflike areas, and pigmentation patterns. Biopsy confirmation is essential for definitive diagnosis, with various biopsy techniques available, including excisional, incisional, shave, and punch biopsies [13]. Dermatoscopy enhances diagnostic accuracy, but histopathological examination remains indispensable, particularly for aggressive-growth subtypes of BCC [25]. Additionally, the use of modern non-invasive imaging techniques, such as reflectance confocal microscopy (RCM), has been shown to significantly improve diagnostic precision, especially in cases where traditional methods yield ambiguous results. RCM allows for real-time, high-resolution imaging of skin at a cellular level, enabling visualization of characteristic BCC features such as dark tumor islands and peripheral clefting, and thus potentially reducing the need for invasive biopsies in select cases [26,27,28,29]. The identification of somatic mutations in genes such as PTCH1, SMO, and TP53 further aids in understanding the molecular pathogenesis of BCC, and in guiding therapeutic strategies [30].

BCC poses challenges in staging due to the lack of a dedicated system. Traditionally grouped with other cutaneous malignancies, BCC’s low metastatic incidence renders conventional tumor, node, and metastasis (TNM) staging irrelevant for localized cases [31]. Cross-sectional imaging for metastasis is infrequent in BCC due to its rarity, but may be considered for extensive cases [31]. Although there is no formal staging system for BCC, stratifying cases based on recurrence risk is clinically crucial. The National Comprehensive Cancer Network (NCCN) Guidelines provide a valuable method for this risk stratification [5,31,32]. A brief summary of the stratification and pathological criteria can be consulted in Tables S1 and S2 in the Supplementary Materials.

Treatment options depend on the size, location, and aggressiveness of the tumor. Decision trees of the treatment are described in Figures S1 and S2 in the Supplementary Materials.

Surgery is the primary treatment for BCC, offering high cure rates and minimal recurrence risk. Mohs micrographic surgery (MMS) is the gold standard for high-risk or recurrent BCCs, providing excellent cure rates and tissue preservation. Excisional surgery is effective for well-defined BCCs, while electrodessication and curettage (ED&C) is simpler, but may have higher recurrence rates (90–95%). In electrodessication, after the visible tumor is removed, an electrode is used to apply an electrical current to the area, destroying any remaining cancerous cells and controlling bleeding by cauterizing the tissue. Reconstructive surgery restores appearance and function post-treatment [13].

While historically a mainstay in most of cancers’ treatment, chemotherapy plays a limited role in the management of BCC [33]. Unlike in other aggressive cancers, where chemotherapy is often utilized as a systemic therapy to target rapidly dividing cells, BCC typically grows slowly and rarely metastasizes. Therefore, chemotherapy is generally reserved for cases of advanced or metastatic BCC, when other modalities, such as surgery or radiation therapy, are not feasible or effective [13,33]. In cases where chemotherapy is considered, the choice of agents and regimens is based on factors such as tumor size, location, extent of disease, and individual patient characteristics. Common chemotherapeutic agents used in BCC treatment include topical agents, as stated previously, such as 5-fluorouracil (5-FU) and imiquimod, which may be applied directly to the skin lesion in cases of superficial or nodular BCC. These medications work by disrupting DNA synthesis and inducing apoptosis in cancer cells [13]. In more advanced or metastatic BCC cases, systemic chemotherapy may be administered orally or intravenously. Chemotherapeutic agents such as cisplatin, carboplatin, and paclitaxel may be used, either as single agents or in combination regimens [34]. These agents target rapidly dividing cancer cells, inhibiting their growth and proliferation [13,33]. While chemotherapy may provide palliative benefits in terms of tumor shrinkage and symptom relief, its efficacy in achieving long-term remission or a cure of BCC is limited [13]. Additionally, chemotherapy is associated with potential side effects, including nausea, vomiting, fatigue, hair loss, and immunosuppression, which can significantly impact patients’ quality of life [13,33].

Radiotherapy serves as a pivotal non-invasive treatment option for BCC, particularly for patients who are not suitable candidates for surgery [35]. This modality is highly effective in treating tumors located in sensitive or cosmetically critical areas, such as the face or ears, where surgical interventions might lead to significant disfigurement or functional impairment [36]. By employing high-energy radiation to damage the DNA of cancer cells, radiotherapy induces cell death and tumor shrinkage [36]. Radiotherapy techniques, including superficial X-ray and electron beam therapy, target tumors precisely, while sparing healthy tissue. Effective for large or recurrent tumors, they achieve cure rates of 85%–95% and can be used as primary or adjuvant therapy after surgery [33]. Despite its efficacy, patients may experience short-term side effects such as skin irritation, redness, and pigmentation changes. Long-term risks include chronic skin changes, scarring, and a slight increase in the risk of secondary cancers [36,37]. Additionally, the treatment regimen requires multiple sessions over several weeks, which can be burdensome for patients. The potential for side effects must be carefully weighed against the therapeutic benefits, particularly in elderly patients or those with significant comorbidities [37,38]. Advancements like intensity-modulated radiation therapy (IMRT) improve tumor targeting and minimize side effects, with ongoing research focused on optimizing protocols for BCC [39].

Additionally, emerging therapies hold promise for improving outcomes and reducing the need for traditional chemotherapy in BCC management [40]. Immunotherapy represents a forefront contender, holding promise in reshaping the standard of care for BCC [41]. In the context of BCC, immune checkpoint inhibitors (ICIs) targeting programmed cell death protein 1 (PD-1) and its ligand (PD-L1) have demonstrated remarkable efficacy [42,43]. Clinical trials evaluating ICIs, such as pembrolizumab and cemiplimab, have shown durable responses and favorable outcomes in patients with advanced or metastatic BCC [44]. These agents unleash the antitumor immune response by blocking inhibitory signals, thereby restoring T-cell-mediated cytotoxicity against tumor cells [13]. Combination therapies incorporating ICIs with other modalities, such as targeted therapy or radiation, are being explored to enhance treatment responses and overcome resistance mechanisms. Additionally, novel immunotherapeutic approaches, including tumor-infiltrating lymphocyte (TIL) therapy and cancer vaccines, hold promise in augmenting the immune response against BCC [41].

Targeted therapy also represents a promising avenue for treatment of BCC. The primary objective of targeted therapy in BCC is to mediate these dysregulated pathways, thereby halting the uncontrolled proliferation of malignant cells. Specifically, the mitogen-activated protein kinase (MAPK) cascade emerges as a pivotal pathway that is frequently implicated in BCC pathogenesis. Mutations in genes such as BRAF, NRAS, and NF1 are prevalent in BCC, mirroring their involvement in melanoma [15,45]. To counteract those mutations, targeted therapies have been developed, focusing on inhibiting key components of the MAPK pathway. Drugs like vemurafenib, dabrafenib, and encorafenib target BRAF, while others like trametinib, cobimetinib, and binimetinib inhibit MEK. Despite their efficacy, these therapies may entail adverse effects, including fatigue, skin toxicity, and other related complications [45,46]. In addition, the hedgehog (Hh) signaling pathway is also crucial in the development of BCC. Mutations in the pathway, particularly in the PTCH1 gene, lead to uncontrolled cell growth. Inhibiting this pathway can effectively stop the growth of BCC. Vismodegib was the first selective Hh inhibitor, approved in 2012. It inhibits the hedgehog signaling pathway, and it was approved for use in patients with locally advanced or metastatic BCC that cannot be treated with surgery or radiation. Phase I and II trials demonstrated the clinical benefits of this drug. The clinical efficacy in the treatment of BCC in two international, multicenter clinical trials showed clinical response ratios of 68.5% and 60.3% [47]. However, safety analyses showed frequent adverse events associated with treatment (98%). Another example is sonidegib. This drug was approved by the FDA on 24 July 2015, to treat patients with locally advanced BCC that has returned after surgery or radiation. In an ongoing multicenter clinical trial, tumor reduction occurred in thirty-eight (58%) of sixty-six patients with locally advanced BCC treated with the lower dose (200 mg), three of which were complete responses. Responses lasted for at least 6 months in nearly 50 percent of these patients [48]. The response rates were similar in those who received the higher dose (800 mg), but patients treated with this dose had worse side effects [48]. One-third of patients in the trial halted treatment because of side effects, which include adverse musculoskeletal-related reactions and a boxed warning for severe birth defects in pregnant women. Sonidegib joins vismodegib in offering new treatment options for patients who are not candidates for surgery or radiation by inhibiting the hedgehog signaling pathway, which is linked to BCC development [48]. However, like melanoma, a major challenge in BCC-targeted therapy lies in the emergence of resistance mechanisms, which can compromise treatment outcomes over time [49,50]. While significant progress has been made, ongoing research efforts are necessary to optimize the use of targeted agents and overcome resistance mechanisms, ultimately improving outcomes for patients with BCC [51].

## 4. Light-Activated Therapies: PDT and PTT Applied to BCC

Photodynamic therapy (PDT) represents a non-invasive treatment modality for BCC, leveraging the synergistic action of photosensitizing agents and light to induce tumor cell death. After the prodrug is metabolized within the cancer cells, light of a specific wavelength (typically red or blue light) is applied to the area. The light energy activates the photosensitizer, generating ROS that cause localized damage and death of the malignant cells [52]. The photosensitizer, typically a porphyrin derivative, selectively accumulates in tumor tissues [53,54].

This achievement, as well as this methodology, was applied to several human carcinomas, one of them being BCC [55]. This fact was crucial to the first FDA approval for PDT, as well as the groundbreaking research made to better understand its underlying mechanisms, such as apoptosis, necrosis, and immunomodulatory effects [56]. PDT has demonstrated efficacy in both primary and recurrent lesions, offering favorable cosmetic outcomes and minimal scarring. Clinical studies have underscored the utility of PDT as a first-line or adjuvant therapy, particularly in superficial and nodular BCC variants.

Mechanistically, PDT-induced tumor cell death involves apoptotic and necrotic pathways, coupled with antiangiogenic effects, thereby inhibiting tumor growth and promoting tissue repair. Moreover, PDT exhibits immunomodulatory properties, eliciting antitumor immune responses and potentially preventing disease recurrence [53]. Its ability to selectively target tumor cells, coupled with favorable cosmetic outcomes and immunomodulatory effects, positions PDT as a valuable therapeutic modality in the armamentarium against BCC.

It is important to acknowledge certain limitations inherent to this approach. One significant challenge is its dependence on the depth of the lesion and the adequacy of light penetration [55,56,57]. Light penetration depends on factors such as the wavelength used, the tissue characteristics, and the presence of blood vessels or other obstructions. Recent studies have highlighted that the effectiveness of PDT may be compromised in deeper lesions, where light penetration is insufficient. This can result in incomplete treatment and recurrence of the tumor. Thus, the limited penetration of light into tissues restricts its use to surface-level or shallow tumors. PTT, especially when using NIR light and AuNPs, can allow for deeper tissue penetration. In addition, the efficacy of PDT is heavily dependent on the presence of molecular oxygen. Hypoxic (oxygen-poor) regions in solid tumors can limit the effectiveness of the therapy because insufficient oxygen can prevent the formation of ROSs, which are critical for inducing cell death. Many tumors, particularly in advanced stages, have areas of low oxygen, reducing the effectiveness of PDT in those regions. Combining the PTT and PDT can reduce the impact of hypoxia, as PTT can compensate for areas where PDT is less effective. Additionally, localized heating from PTT could improve blood flow, enhancing oxygen delivery to the tumor, and thereby improving PDT’s efficacy. Moreover, since the success of PDT hinges on both the photosensitizer’s activation and the effective depth of light penetration, variability in tumor thickness and structure can lead to inconsistent treatment outcomes, especially in thicker or more aggressive forms of BCC. For example, tumors located in areas with thicker skin or higher vascularity may respond less effectively to the treatment. Additionally, certain skin types, particularly those with darker pigmentation, may absorb more light energy in the superficial layers, reducing the amount of light that reaches deeper tissues. In addition, one of the drawbacks for patients undergoing PDT is the risk of prolonged photosensitivity. After treatment, patients may become highly sensitive to sunlight and other strong light sources for an extended period, sometimes lasting weeks or even months. Thus, this underscores the necessity for careful patient selection and treatment planning to optimize therapeutic outcomes [55,56,57].

Currently, the main photosynthesizer used for BCC treatment is PpIX. This was made possible after the topical application of a prodrug in the BCC lesions, such as 5-aminolevulinic acid (ALA) or methylaminolevulinate (MAL). The significant variance in absorption of these components by BCC cells when compared to healthy tissue is a key component to its efficacy. When absorbed by BCC cells, these prodrugs are metabolized in the mitochondria via a heme-biosynthetic pathway, and are then converted to PpIX, which is the active photosensitizer; its ability to absorb light and produce a triplet-excited state within electrons causes the production of ROS when in the presence of oxygen, which leads to cell apoptosis and necrosis [55].

While emerging therapies have shown considerable promise in managing BCC, the advent of photothermal therapy (PTT) with gold nanoparticles represents a significant leap forward, combining the precision of nanotechnology with therapeutic efficacy to potentially revolutionize BCC treatment.

A different approach to the treatment of this disease is photothermal therapy (PTT). It is a treatment based on nonionizing radiation in combination with a light-sensitive thermal nanoplatform named the photothermal enhancer [58,59,60]. PTT relies on the irradiation of nanomaterials by a light source, commonly in the near-infrared (NIR) or visible spectrum. The “biological window or BW” (or optical/therapeutic window) includes radiation with a wavelength ranging from 650–1300 nm (BW I and BW II). Here, the light can penetrate more deeply into the tissue due to the absence of absorptions by other biological species in that range. Gold is one of the nanomaterials often used for this due to its excellent photothermal properties.

Gold (Au), one of the noble metals, has been characterized by its resistance to corrosion and oxidation. Colloidal Au was documented in the Middle Ages as a substance for treatment and diagnosis of diseases. Inspired by the early discovery of the bacteriostatic properties [61], gold compounds were eventually applied for many treatments, including rheumatoid arthritis, tuberculosis, and certain cancers [62]. Recent advancements have recognized the use of Au in the therapeutic delivery of drugs, or as a therapeutic modality. For example, colloidal Au is covalently linked onto adenoviral vectors for selective cancer targeting, and it induces hyperthermia by application of a NIR laser light [63].

The collective oscillation of free electrons in AuNPs (the SPR) results in efficient conversion of light energy into heat. This localized heating effect induces photothermal ablation of cancerous cells, leading to tumor destruction. Furthermore, AuNPs exhibit excellent stability and biocompatibility, ensuring favorable pharmacokinetics in vivo. Another advantage is that AuNPs can be synthesized with a certain control over their size and shape, allowing customization of their optical and thermal properties. They can also be functionalized with targeting ligands, such as antibodies or peptides, to enhance their specificity towards cancer cells. Additionally, surface modification with biocompatible coatings improves stability and biocompatibility, facilitating their use in biological systems [58,64]. Their inert nature and biocompatibility make them suitable candidates for biomedical applications, including cancer therapy [58].

Gold nanoparticles (AuNPs) are typically administered via intravenous injection. Surface modifications, such as PEGylation, can enhance circulation time and a particle’s stability. In photothermal therapy (PTT), AuNPs are activated using near-infrared (NIR) light, providing localized heating for precise tumor ablation. Direct intratumoral injection ensures high local concentration, improving therapeutic outcomes while minimizing systemic side effects [65,66,67]. Functionalization with targeting ligands enhances specificity towards cancer cells, optimizing treatment efficacy [68,69].

The interaction between AuNPs and NIR laser radiation induces an increase in the temperature inside the tissues, which is called hyperthermia. This physical mechanism of action consists in converting the incident energy from photons absorbed by NPs, at a specific wavelength [70], making use of a localized SPR (LSPR) effect. The transformation of the ground singlet state photons into an excited singlet state then leads to relaxation, a nonradiative form of decay, whereby a return to the ground state is mediated by collisions between the excited photothermal agents and their surrounding molecules [70]. The microenvironment of the nanoparticles is thus heated. Consequently, increased kinetic energy leads to heating of the surrounding microenvironment, which is called exalted hyperthermia (>42 °C) [70].

In terms of PTT and BCC, Pesnel and collaborators reported the first proof of concept for PTT/AuNPs applied to BCC. The study demonstrated that NIR laser stimulation of AuNPs induced significant exalted hyperthermia within minutes, even at a low power of 1 W. Preclinical feasibility and safety were confirmed on small surface lesions (50 mm^3^), paving the way for studying PTT on larger tumor volumes (>50 mm^3^) [70].

Moreover, recent studies have shown that combining PTT with PDT may enhance treatment efficacy. PDT utilizes photosensitizing agents that, when activated by light, produce ROS, leading to tumor cell death. The integration of PDT with PTT could potentially increase oxygen supply and improve therapeutic outcomes, particularly for BCC [71]. Additional research on AuNPs functionalized with glucose also demonstrated the efficacy in cancer cell targeting, internalization, and intracellular trafficking [72]. Specifically, these AuNPs exhibited superior internalization into cancer cells, attributed to glucose-mediated targeting of overexpressed glucose transporters (GLUTs), particularly GLUT1 and GLUT3 [50]. Mechanistic insights revealed clathrin-dynamin-dependent endocytosis and lysosomal exocytosis pathways in intracellular trafficking, and excretion and preclinical validation in a murine melanoma model demonstrated significant antitumor activity of those AuNPs, with a favorable safety profile [58,72]. Future research may explore combination therapies and diagnostic applications to maximize the therapeutic benefits of nanotheranostics in BCC management but also in other cancers [33,58,72,73].

In another study, a novel NIR light-responsive hydrogel-forming long needle loaded with Au nanorods showed a potential photothermal effect for localized treatment of BCC [74]. The formulation was prepared with Gantrez^®^ S-97 and poly(ethylene glycol) (PEG) with 200 Da, and it was characterized in terms of swelling, insertion, and mechanical properties. The target SPR band at 785 nm belongs to the first biological window (BW I), which includes a range of wavelengths (approximately 650–950 nm) that are relatively less absorbed and scattered by biological tissues, allowing a deeper tissue penetration. This specific SPR matches the output of the laser used for treatment, ensuring optimal absorption and conversion of light into heat by the gold nanorods. Consequently, the needles rapidly reached a temperature of approximately 50 °C upon exposure to a laser with 785 nm wavelength, at a power of 60 mW.

## 5. A Special Focus on This New Golden Era

Over the past 30 years, nanosized materials have emerged as promising tools for tumor targeting, diagnosis, and therapy, presenting a viable alternative to conventional treatment modalities. These nanomaterials, particularly nanoparticles (NPs), offer several advantages over traditional therapies [75,76,77,78,79]. One significant benefit is their ability to extend the circulation time of therapeutic agents in the bloodstream, thereby enhancing drug delivery efficiency [80]. Moreover, NPs can accumulate selectively at tumor sites due to their unique structural properties, such as surface ligands, and their small size, which facilitates permeation through blood vessels [58,72,81]. In addition to drug delivery applications, nanosized materials have also garnered attention for their utility in PTT. AuNPs have demonstrated effectiveness in this context [40].

This minimally invasive approach holds promise for the treatment of various cancers, particularly superficial malignancies like non-metastatic melanoma [74,80,81,82]. Studies suggest that this therapy yields superior results when the energy source used is in the NIR region, within the optimal therapeutic window (BW 650–1300 nm). In this BW, the light can penetrate deeper into tissues once it is less absorbed and scattered. Moreover, if the AuNPs present SPR in this region, it is more likely that the light absorption will be enhanced at the targeted region where the AuNPs are accumulated, minimizing adverse effects on surrounding healthy tissues. However, the effectiveness of this approach depends on the size and shape of the NPs, as well as the characteristics of the surrounding environment. The effectiveness for deeper cancers is limited by the penetration’s depth of the laser [18,64].

### 5.1. Mechanisms of Action

AuNPs play a central role in PTT, harnessing their unique optical properties to absorb NIR light and convert it into heat through SPR. This localized hyperthermia selectively destroys cancer cells while sparing healthy tissue, showcasing the potential of nanomaterials in improving therapeutic outcomes. The mechanism of action of PTT with AuNPs involves multiple intricate steps. Firstly, AuNPs are designed to specifically target cancer cells, often through surface functionalization with specific ligands such as antibodies or peptides. Once administered, these functionalized AuNPs circulate in the bloodstream, exploiting tumor-specific vascular abnormalities to potentially accumulate within the tumor microenvironment [4,15].

Understanding the permeation behavior of AuNPs is crucial for optimizing their efficacy in PTT. Several advanced techniques are employed to study the permeation and distribution of AuNPs in biological tissues. These methods include Transmission Electron Microscopy, which provides high-resolution imaging of AuNPs, allowing researchers to track their location at the cellular and subcellular levels. In addition, Confocal Laser Scanning Microscopy can be used to visualize their distribution within tissues in three-dimensions, helping to understand how nanoparticles interact with the extracellular matrix and other components of the tumor microenvironment. Quantitatively, Inductively Coupled Plasma Mass Spectrometry can be employed to quantify the amount of AuNPs in biological tissues by measuring the gold content in different organs or tissue sections. Finally, in vivo imaging techniques, such as MRI, CT, and PET scans, can be used to monitor the distribution of AuNPs in live animals, providing real-time information on how AuNPs permeate tissues and accumulate at the tumor site over time.

**Figure 4:**
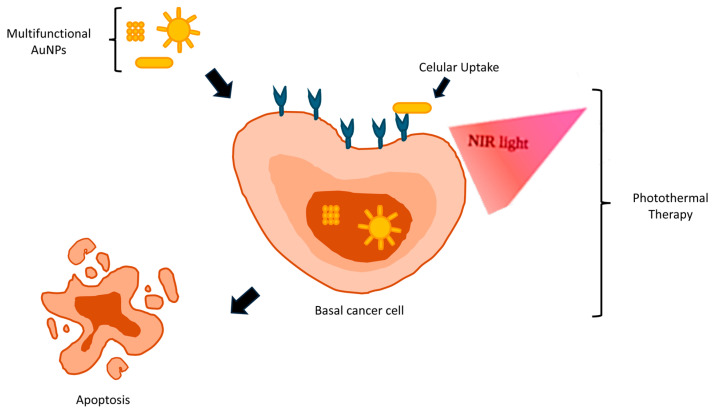
Photothermal therapy (PTT) in BCCs.

Upon NIR irradiation, as illustrated in Figure 4, AuNPs efficiently absorb light energy, elevating local temperatures within the tumor site. This localized hyperthermia might trigger a cascade of cellular events, including protein denaturation, membrane disruption, and, ultimately, apoptotic or necrotic cell death. Importantly, the selectivity of PTT ensures enhanced targeting of cancer cells while minimizing the damage to adjacent healthy tissues, thereby reducing treatment-related side effects [4,18].

The synergy between nanomaterials and PTT heralds a new era in cancer therapeutics, offering unparalleled precision and efficacy in tumor destruction. Ongoing research efforts continue to refine and optimize this innovative approach, with promising results in preclinical and clinical studies [40]. Recent studies have shed light on additional aspects of PTT with AuNPs. Activation tests of HAOA-coated AuNPs with a laser revealed intriguing phenomena within the tumor microenvironment [58]. The in vitro activation temperature was found to be approximately 42–45 °C, emphasizing the importance of avoiding high temperatures [58,83,84].

Since the early 20th century, additional research has been conducted in which hyperthermia was carefully applied to cancerous tumors, maintaining tissue temperatures around 42–45 °C. Early pioneers, like William Coley in 1893, focused on stimulating immune responses to fight cancer, laying the groundwork for modern therapeutic approaches. Photothermal therapy (PTT) has roots in ancient history, where it was known by various names. The therapeutic benefits of light, sourced from the sun or other UV or IR light sources (heliotherapy), have been recorded since Ancient Egypt, in the Ebers papyrus [85], and by Herodotus in Greece, where it was used to treat various pathologies [86]. In 1903, the Nobel Prize in Medicine was awarded to Niels Ryberg Finsen, a Danish physician, for his contributions to the treatment of various illnesses using concentrated light radiation [87].

### 5.2. Off-Label Uses of Light-Activated Therapies

Photothermal therapy (PTT) has shown promising off-label uses across various medical fields beyond its primary application in oncology. In infectious diseases, PTT has been explored for its antibacterial effects, demonstrating efficacy in treating bacterial infections by utilizing photothermal-responsive nanomaterials to eradicate pathogens [66,88]. Additionally, PTT has been applied in dermatology for conditions like acne vulgaris, where nanomedicine approaches have been investigated for their therapeutic potential, providing an alternative to traditional treatments [89]. In neurology, PTT is being studied for its potential in treating neurological diseases, leveraging the localized heating effects to target specific neural tissues and manage neurological disorders [90].

Ophthalmology has also benefited from PTT applications, where low-level laser therapy has been employed to stimulate healing and restore function in various ocular conditions [91]. Cardiovascular research has explored PTT for treating cardiovascular diseases, utilizing laser-induced photothermal interactions to target and reduce atherosclerotic plaques [92]. Furthermore, PTT has been investigated in pain management, where its ability to provide localized heating offers a novel approach to alleviating pain without systemic side effects [93]. In the realm of cosmetic procedures, PTT has gained attention for non-invasive fat reduction and skin tightening, offering patients cosmetic benefits with minimal recovery time compared to traditional surgical methods [94,95]. These diverse applications highlight the versatility and potential of PTT as a multifaceted therapeutic tool in modern medicine.

The underlying rationale for these off-label uses of PTT, especially in dermatology and related fields, can be correlated with the treatment of BCC due to the localized, controlled heating mechanism of PTT. The selective targeting of abnormal tissues in BCC through photothermal ablation parallels its use in dermatological conditions like acne vulgaris, where inflammation and bacterial activity are key components. In both cases, the ability to localize treatment using in situ administration of photothermal agents and NIR (low-energy radiation) while sparing surrounding healthy tissue is crucial. Similarly, in cardiovascular applications, where PTT targets atherosclerotic plaques, the precision of photothermal interactions aligns with the need for selective tumor destruction in BCC, especially for aggressive or deeply seated lesions [66,67,96,97]. By inducing localized hyperthermia, PTT can trigger apoptotic and necrotic cell death pathways in BCC, which reflects the broader biological mechanisms used in off-label contexts.

Moreover, there is a known correlation between UVB radiation and the metabolism of cholecalciferol (vitamin D3), which then correlates with the capability of osteoblasts to synthesize the collagen matrix. Collagen is crucial for the structural integrity and strength of bones, providing the framework upon which minerals such as calcium phosphate are deposited, which is essential for bone density and strength. In addition to its role in bone health, collagen is also integral to the skin’s structure, supporting elasticity and resilience. Therefore, understanding how UVB radiation impacts collagen synthesis by osteoblasts can provide insights into both bone health and skin diseases [98].

In clinical contexts, this knowledge has implications for treating various conditions. For instance, psoriasis, an autoimmune skin disorder, has shown positive responses to treatments that influence vitamin D metabolism, potentially due to ALA-PDT anti-inflammatory effects [99]. Similarly, conditions like Hirsutism, characterized by abnormal hair growth in androgen-sensitive areas, have traditionally relied on mechanical hair removal methods. Studies employing ALA-PDT showed a 75% reduction over the course of 12 months [99]. This effect might be related to the local inflammation, as well as the cytotoxic effect of the compound; however, further studies are needed [98].

Additionally, PDT can be a very interesting approach for the treatment of Crohn’s disease. Crohn’s disease is an autoimmune inflammatory disease in the gastrointestinal tissue with symptoms that gravely impact quality of life. In a rat model, ALA-PDT was used, and the results demonstrated its anti-inflammatory potential as a modulator of immune response [98].

### 5.3. Clinical Trials of AuNPs, PTT, and PDT

The FDA has approved a limited number of AuNP-based technologies for both diagnostic and therapeutic purposes [100]. The efficacy and safety of AuNPs in cancer treatment are influenced by factors such as particle size, morphology, environmental conditions, and production methods [101,102,103].

One example of a clinical trial was conducted by AstraZeneca in collaboration with Cytimmune, and it focuses on Aurimune (CYT-6091), where AuNPs act as carriers for recombinant human tumor necrosis factor alpha (rhTNF) [104]. This approach disrupts tumor blood vessels, allowing chemotherapeutic agents to penetrate tumors effectively, and has demonstrated the safe delivery of higher doses of rhTNF compared to traditional methods [105]. The addition of a PEG layer reduces nanoparticle uptake by the mononuclear phagocytic system (MPS), enhancing their accumulation in tumor tissues [104].

Another example is Aurolase^®^, developed by Nanospectra Biosciences, which utilizes silica–gold nanoshells coated with PEG. These nanoshells are designed to thermally ablate solid tumors upon exposure to NIR light [106]. Clinical trials have investigated Aurolase^®^ for primary and metastatic lung tumors (NCT01679470) and refractory head and neck cancers (NCT00848042), highlighting its potential for targeted cancer treatment with minimal off-target effects [104].

Other ongoing clinical trials are exploring novel AuNP-based theranostics, including NU-0129. NU-0129 combines gold nanoparticles with small interfering RNAs (siRNAs), targeting BCL2L12 for potential antineoplastic activity (NCT03020017) [107].

Other trials investigate this strategy using CNM-Au8, including a trial focused on safety, tolerability, and pharmacokinetics (reference NCT02755870), as well as its application in neuromyelitis [108]. This strategy resulted in improved progressive vision and cognition in patients with relapsing multiple sclerosis (clinical trial named VISIONARY-MS, with reference NCT03536559). Other clinical trials (safety and/or efficacy trials) involving gold have been developed, such as REPAIR-ALS (NCT03843710), The HEALEY ALS Platform Trial (NCT04414345), the HEALEY ALS Platform Trial—Master Protocol (NCT04297683), and RESCUE-ALS (NCT04098406), all for amyotrophic lateral sclerosis; clinical trials for multiple sclerosis like REPAIR-MS (NCT03993171 and NCT04081714); and trials or for Parkinson’s disease such as REPAIR-PD (NCT03815916).

Additionally, innovative diagnostic approaches utilizing AuNPs are being studied to detect gastric diseases (NCT01420588) and pulmonary arterial hypertension (NCT02782026) through breath and electronic nose sensors, respectively [104].

All clinical trials, detailed below in Table 1, underscore the expanding role of AuNPs in advancing therapeutic interventions and diagnostic capabilities across various medical conditions, offering promise for enhanced treatment efficacy and improved patient outcomes in the future [104].

## 6. Conclusions and Future Perspectives

BCC remains the most common form of skin cancer, with increasing incidence rates globally. Traditional treatments, including surgical excision, electrodessication and curettage, and radiation therapy, are effective, but come with limitations, particularly in advanced or recurrent cases.

Light-activated therapies present an innovative and promising approach to BCC treatment. In the case of PTT, advances have been noticed due to the unique optical properties of AuNPs to selectively target and destroy cancer cells with minimal damage to surrounding healthy tissue. Nevertheless, several challenges need to be addressed. Ensuring the biocompatibility and safety of AuNPs remains an important concern. Consequently, long-term toxicity studies and a thorough, full investigation through extensive in vitro and in vivo studies are required. Furthermore, achieving precise delivery of AuNPs to tumor sites without affecting healthy tissues is critical. Thus, developing effective targeting mechanisms, such as functionalization with tumor-specific ligands, is essential for improving the specificity. In terms of large-scale production, it is crucial to standardize the synthesis of AuNPs, as well as their size, shape, and surface modification, in order to ensure consistent and reproducible results across different studies and clinical applications. Furthermore, also related to AuNPs production, the complex manufacturing processes may limit the widespread adoption of this technology. Therefore, efforts to reduce production costs and improve scalability are needed to make this strategy more accessible. On the other hand, the effectiveness of PTT is often limited by the depth of light penetration in tissue, which can restrict its use to superficial or accessible tumors. Strategies to enhance light delivery to deeper tissues are necessary for broader applicability. Finally, integrating PTT with other treatment modalities, such as chemotherapy, immunotherapy, or radiation therapy, could offer synergistic effects, maximizing therapeutic outcomes and reducing the likelihood of resistance.

Thus, continued research and development in this area hold the potential to revolutionize the management of BCC, offering more effective, non-invasive, and targeted treatment options for patients.

## Figures and Tables

**Table 1:** Clinical trials of AuNPs. Information adapted from references [99,102,103].

Name	Materials	Applications	Clinical Trials Identifier
AuroLase	Silica–gold nanoshells coated with PEG	Laser responsive thermal ablation of solid tumor: head/neck cancer, primary and/or metastatic lung tumors.	NCT00848042 NCT01679470
AuroLase	Silica–gold nanoshells coated with PEG	RI/US fusion imaging and biopsy of the prostate, head, neck and lungs combined with nanoparticle direct focal therapy for ablation of prostate tissue.	NCT02680535
NU-0129	Spherical nucleic acid (SNA) gold nanoparticles	Targeting BCL2L12 in recurrent glioblastoma multiforme or gliosarcoma patients.	NCT03020017
CNM-Au8	Gold nanocrystals	Evaluation of safety, tolerability, and pharmacokinetics of CNM-Au8 in healthy male and female volunteers (future experimental oral therapy for amyotrophic lateral sclerosis)	NCT02755870
Gold nanoparticles	Gold nanoparticles	Sensors functionalized with Au nanoparticles ^1^. Organic functionalized nanoparticles ^2^. Detection of gastric lesions ^3^. Exhaled breath olfactory signature of pulmonary arterial hypertension ^4^.	NCT01420588 ^1,2,3^ NCT02782026 ^4^
Silica–gold nanoparticles	Silica–gold nanoparticles	Plasmonic photothermal therapy of flow-limiting atherosclerotic lesion.	NCT012700139

The following associations are made between the discussed topics and the corresponding clinical trials, indicated by the numbered references: (a) Sensors functionalized with Au nanoparticles—Clinical Trial: NCT01420588 (1, 2, 3). (b) Organic functionalized nanoparticles—Clinical Trial: NCT01420588 (1, 2, 3). (c) Detection of gastric lesions—Clinical Trial: NCT01420588 (1, 2, 3). (d) Exhaled breath olfactory signature of pulmonary arterial hypertension—Clinical Trial: NCT02782026 (4).

## Data Availability

Not applicable.

## Supplementary Materials

The following supporting information can be downloaded at: https://www.mdpi.com/article/10.3390/ijms252111494/s1. References [5,6,13,15,18,31,32,109,110,111,112,113] are also mentioned in Supplementary Materials.

## Abbreviations

ALA, aminolevulinic acid; Au, gold; AuNPs, gold nanoparticles; BCC, basal cell carcinoma; BCNS, basal cell naevus syndrome; BRAF, a gene that is part of the RAF family of serine/threonine protein kinases; BW, biological window; ED&C, electrodessication and curettage; GS, Gorlin syndrome; GLUTs, glucose transporters; Hh, hedgehog; ICIs, immune checkpoint inhibitors; IMRT, intensity-modulated radiation therapy; MAL, methylaminolevulinate; MAPK, mitogen-activated protein kinase; MMS, Mohs micrographic surgery; MPS, mononuclear phagocytic system; NF1, neurofibromin 1 gene; NIR, near-infrared radiation; NRAS, a member of the Ras family of GTPases; PD-1, cell death protein 1; PD-L1, cell death protein 1 ligand; PDT, photodynamic therapy; PEG, poly(ethylene glycol); PTT, photothermal therapy; rhTNF, recombinant human tumor necrosis factor alpha; ROS, reactive oxygen species; SMO, sonic hedgehog signaling molecule; SUFU, suppressor of fused; SPR, surface plasmon resonance; TIL, tumor-infiltrating lymphocyte; XP, xeroderma pigmentosum.
