# Supplementary material for: Combining Phototherapy and Gold-Based Nanomaterials: A Breakthrough in Basal Cell Carcinoma Treatment
Source: Int J Mol Sci. 2024 Oct 26;25(21):11494. doi: 10.3390/ijms252111494 (PMC11545837; doi:10.3390/ijms252111494)
Supplement: Supplementary file 1 [file ijms-25-11494-s001.zip › ijms-3158672-supplementary.pdf]

International Journal of *Molecular Sciences*

MDPI

Review

Combining Phototherapy and Gold-Based Nanomaterials: A Breakthrough in Basal Cell Carcinoma Treatment

Karolyne Silva Baioco [1], Raquel Pereira [1], Tânia Ferreira-Gonçalves [1,2], João M. P. Coelho [2], Maria Manuela Gaspar [1,2] and Catarina Pinto Reis [1,2,*]

[1] Research Institute for Medicines (iMed.ULisboa), Faculty of Pharmacy, Universidade de Lisboa, Av. Professor Gama Pinto, 1649-003 Lisboa, Portugal; silvabaiocokarolyne@gmail.com (K.S.B.); ana.raquel1705@gmail.com (R.P.); tsgoncalves@ciencias.ulisboa.pt (T.F.-G.); manuelagaspar@campus.ul.pt (M.M.G.)

[2] Instituto de Biofísica e Engenharia Biomédica (IBEB), Faculdade de Ciências, Universidade de Lisboa, Campo Grande, 1749-016 Lisboa, Portugal; jmcoelho@ciencias.ulisboa.pt

* Correspondence: catarinareis@ff.ulisboa.pt; Tel.: +351-217-946-400 (ext. 14244)

Cancer is a global problem that accounts almost 13% of deaths worldwide. While often less aggressive than other types of skin cancer such as melanoma, Basal Cell Carcinoma (BCC) remains a major challenge due to its rising occurrence and impact on quality of life, emerging as a global public health concern [6].

The National Comprehensive Cancer Network (NCCN) Guidelines offer a valuable stratification method, integrating clinical and pathologic factors and expert opinions from diverse fields. This stratification significantly influences treatment recommendations, providing a crucial framework for BCC management [5,31,32]. Incorporating advanced diagnostic methods could also complement this stratification by providing more precise assessments of tumor margins and behavior, further enhancing clinical decision-making [5,18].

Table 1 and Table 2 depict the TMN stratification and pathological criteria, respectively.

Table S1. TMN stratification and risk associated. Information adapted from Basset-Seguin and Herms [13].

**Clinical Criteria**	**Low Risk**	**High Risk [1]**
Location and size	Area L [2] ≤ 20 mm (maximum clinical diameter)<br>Area M [3] ≤ 10 mm (maximum clinical diameter)	Area L [2] > 20 mm (maximum clinical diameter)<br>Area M [3] > 10 mm (maximum clinical diameter)<br>Area H [4]
Borders	Well defined	Poorly defined
Primary vs recurrent	Primary	Recurrent
Immunosuppression	No	Yes
Site of prior radiotherapy	No	Yes

**Margins**		
Histological margins	Not involved (≥ 1 mm)	Involved (0 mm) or histologically close (< 1 mm)

[1] One or more criteria equals high risk, unless stated differently in the summary of the recommendations, or in an explanatory note. [2] Area L = trunk and extremities but excluding hands, nail units, genitals, pretibia, ankles and feet. [3] Area M = cheeks, forehead, scalp, neck and pretibial. [4] Area H = 'mask areas' of face [central face, eyebrows, periorbital, nose, lips (cutaneous and vermilion), chin, mandible, preauricular, postauricular, temple, ears]; genital areas; hands, nail units, ankles and feet, but excluding the eyelid.

Table S2. Pathological Criteria and Risk associated. Information adapted from [5,31,32].

Pathological criteria / BCC stage	Low Risk	High Risk [1]
Growth pattern	Nodular or superficial	Infiltrative (infiltrating, morphoeic, micronodular)
Differentiation: Basosquamous	Absent	Present (with or without lymphovascular invasion)
Level of invasion [2]	Dermis, subcutaneous fat	Beyond subcutaneous fat
Depth (thickness)	≤ 6 mm	> 6 mm
Perineural invasion	Absent	Present
Pathological TNM stage	pT1 ≤ 20 mm (maximum diameter)	pT2 > 20 mm but ≤ 40 mm (maximum diameter)<br>pT3 > 40 mm (maximum diameter), or upstaged [3] pT1 or pT2, or minor bone invasion pT4 major bone invasion

TNM, Tumour–Nodes–Metastasis. [1] For tumors < 6 mm in size without other high-risk features, standard surgical excision may be considered if a ≥ 4 mm clinical surgical margin can be obtained without significant anatomical or functional distortions. [2] A named nerve or a diameter ≥ 0 1 mm or beyond the dermis. [3] T1 and T2 can be upstaged to T3 by the presence of one or more high-risk clinical or pathological factors comprising specifically defined perineural invasion or deep invasion representing either a tumor thickness or depth > 6 mm and/or invasion beyond or further than the subcutaneous fat.

An early detection and treatment are crucial for preventing the cancer from spreading and causing further damage.

Treatment options depend on the size, location, and aggressiveness of the tumor [5,6,18,31,32]. Figures S1 and S2 describe the decision trees for low and high-risk BCC treatment strategies, respectively.

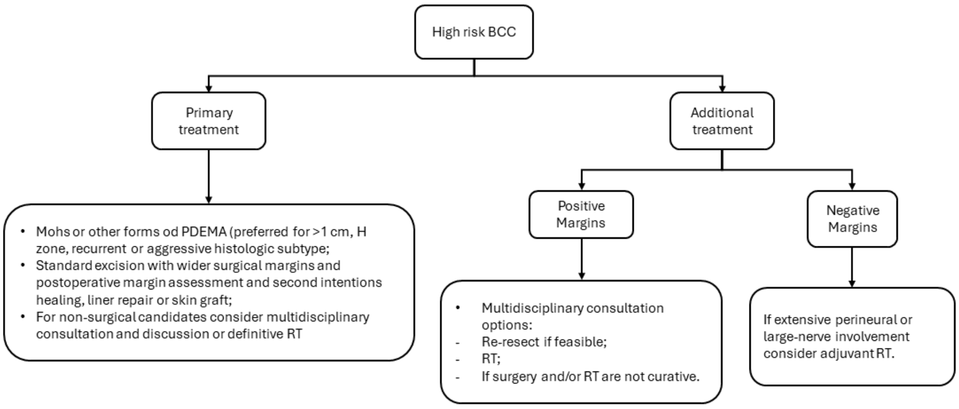

Figure S1. Low risk BCC treatment strategies. Information adapted from [15,109,110].

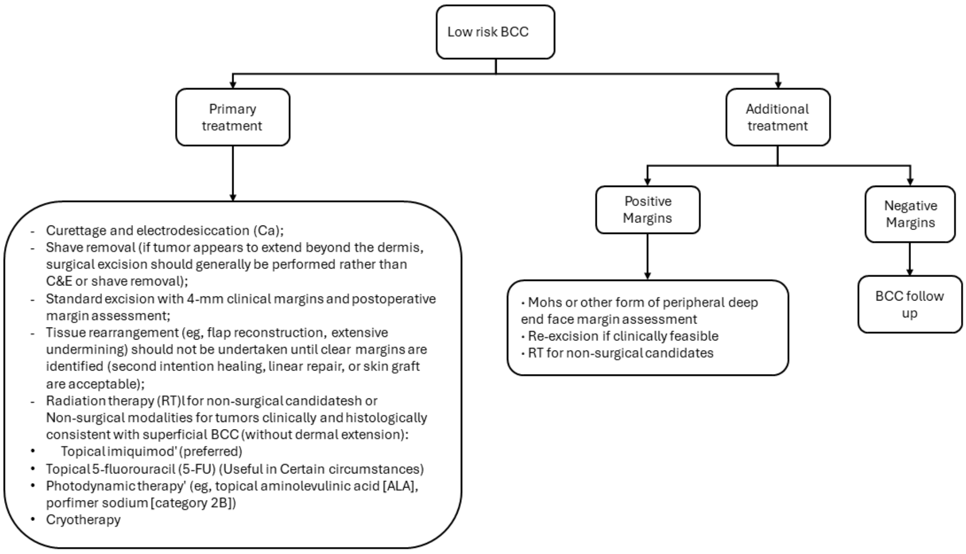

Figure S2. High risk BCC treatment strategies. Information adapted from [13,111–113].

References

5. Schmults, C.D.; Blitzblau, R.; Aasi, S.Z.; Alam, M.; Amini, A.; Bibee, K.; Bordeaux, J.; Chen, P.-L.; Contreras, C.M.; DiMaio, D.; et al. Basal Cell Skin Cancer, Version 2.2024, NCCN Clinical Practice Guidelines in Oncology. *J. Natl. Compr. Canc. Netw.* **2023**, *21*, 1181–1203, doi:10.6004/jnccn.2023.0056.

6. McDaniel, B.; Badri, T.; Steele, R.B. Basal Cell Carcinoma; StatPearls: Treasure Island, FL, USA, 2024. https://www.ncbi.nlm.nih.gov/books/NBK482439/

13. Basset-Seguin, N.; Herms, F. Update in the Management of Basal Cell Carcinoma. *Acta Derm. Venereol* .**2020**, *100*, adv00140, doi:10.2340/00015555-3495.

15. Bakshi, A.; Chaudhary, S.C.; Rana, M.; Elmets, C.A.; Athar, M. Basal Cell Carcinoma Pathogenesis and Therapy Involving Hedgehog Signaling and Beyond. *Mol. Carcinog.* **2017**, *56*, 2543–2557. https://doi.org/10.1002/mc.22690.

18. López-Gómez, M.; Malmierca, E.; Górgolas, M.; Casado, E. Cancer in Developing Countries: The next Most Preventable Pandemic. The Global Problem of Cancer. . *Crit. Rev. Oncol. Hematol* **2013**.88, 117-122. https://doi.org/10.1016/j.critrevonc.2013.03.011.
31. Nasr, I.; McGrath, E.J.; Harwood, C.A.; Botting, J.; Buckley, P.; Budny, P.G.; Fairbrother, P.; Fife, K.; Gupta, G.; Hashme, M.; et al. British Association of Dermatologists Guidelines for the Management of Adults with Basal Cell Carcinoma 2021*. *Br. J. Dermatol.* **2021**, *185*, 899–920. https://doi.org/10.1111/bjd.20524.
32. NCCN GUIDELINES FOR PATIENTS® 2022 Basal Cell Skin Cancer. Available online: https://www.nccn.org/patients/guidelines/content/PDF/basal-cell-patient-guideline.pdf (Accessed date: 8 june 2024).
109. Zhang, H.; Hu, L.; Cheng, M.; Wang, Q.; Hu, X.; Chen, Q. The Hedgehog Signaling Pathway Promotes Chemotherapy Resistance via Multidrug Resistance Protein 1 in Ovarian Cancer. *Oncol Rep* **2020**, *44*, 2610–2620, doi:10.3892/or.2020.7798.
110. Shah, V. V.; Aldahan, A.S.; Mlacker, S.; Alsaidan, M.; Samarkandy, S.; Nouri, K. 5-Fluorouracil in the Treatment of Keloids and Hypertrophic Scars: A Comprehensive Review of the Literature. *Dermatol. Ther.* **2016**, *6*, 169–183, doi:10.1007/s13555-016-0118-5.
111. Narayanan, K.; Hadid, O.H.; Barnes, E.A. Mohs Micrographic Surgery versus Surgical Excision for Periocular Basal Cell Carcinoma. *Cochrane Database of Systematic Reviews* **2014**, *2014*, doi:10.1002/14651858.CD007041.pub4.
112. Chen, J.T.; Kempton, S.J.; Rao, V.K. The Economics of Skin Cancer: An Analysis of Medicare Payment Data. *Plast. Reconstr. Surg. Glob. Open* **2016**, *4*, e868, doi:10.1097/GOX.0000000000000826.
113. Wahid, M.; Jawed, A.; Mandal, R.K.; Dar, S.A.; Khan, S.; Akhter, N.; Haque, S. Vismodegib, Itraconazole and Sonidegib as Hedgehog Pathway Inhibitors and Their Relative Competencies in the Treatment of Basal Cell Carcinomas. *Crit. Rev. Oncol. Hematol.* **2016**, *98*, 235–241, doi:10.1016/j.critrevonc.2015.11.006.
